# Best Approach for Low-Risk Superficial Bladder Cancer

**DOI:** 10.1100/tsw.2006.407

**Published:** 2006-10-23

**Authors:** Eduardo Solsona

**Affiliations:** Service of Urology, Instituto Valenciano de Oncologia, Valencia, Spain

**Keywords:** bladder tumor, superficial, non-muscle invasive, low-risk, chemotherapy

## Abstract

In order to create better criteria for different grades of superficial bladder tumor, the WHO and ISUOP develop a new classification in 1998, which was modified in 2004. Although the new classification might be more reproducible, it has not yet been widely accepted. The low risk groups include patients with single Ta, G1, ≤3cm diameter tumors. This group has high recurrence and low progression rates. The standard treatment is the complete resection and postoperative single immediate instillation of whatever chemotherapy agent should be considered.

## INTRODUCTION

The classification of superficial bladder tumor or better-known nonmuscle-invasive bladder tumors is based on two pillars: (1) the clinical stage following the TNM classification differentiating between Ta, which includes tumors not invading the submucosa, and T1, which invades the submucosa, but not the muscularis propria; and (2) the histological grade that refers to the 1973 WHO grading system based on the microscopic appearance of cancer cells. However, the major limitation of this classification is the vague definition and the lack of specific histological criteria without a reliable inter- and intraobserved correlation[1]. Attempting to create better criteria for different grades in 1998, the WHO and ISUOP developed a new classification, which was modified in 2004[2,3] and included papilloma. G1 tumors were reclassified as papillary urothelial neoplasms of low malignant potential (PUNLMPs) or true low-grade tumors, G2 tumors were also reclassified as low grade and high grade according to specific cytological and histological architectural criteria, and G3 tumors remained as high-grade carcinomas. Until the new classification system is validated, we can use both former and new classifications, and the risk groups are prevailing.

Although the new classification might be more reproducible, it has not yet been widely accepted. Currently, the risk groups of nonmuscle-invasive bladder tumors are under revision, as the classic risk groups are based on the combination of recurrence and progression risks, and many times, both are not parallel in individual and collective bases[4]. Assuming these limitations and following the European Guidelines definition, the low-risk group includes patients with single, Ta, G1, ≤3 cm diameter tumors[5]. This group is characterized by a mean recurrence and progression percentage of 36.5% (range 29–48%) and of 3.3% (range 0–7%) at a 5-year follow-up, respectively[6,7]. With these biological characteristics, patients included in the low-risk group have an excellent biological behavior with no life-threatening, but with moderate, recurrence rate, which compromises their quality of life, requiring many transurethral resections and cystoscopies during follow-up.

As a whole, in patients with TaG1 tumors, the recurrence rate depends on the follow-up length; 40 and 71% at 3 and 5 years of follow-up[8,9]. Also, this recurrence rate is related to multiple tumors, recurrence at 3 months, and previous recurrence rate[10]. All these variables define a subgroup of patients with high recurrence rate, regardless of the therapy applied.

## INCIDENCE

Nonmuscle-invasive bladder cancer comprises the most prevalent group of bladder tumors, around 75%, and the Ta category represents almost 50% of the newly diagnosed nonmuscle-invasive tumors and 70% of these were low grade[8]. In the Millan et al. series[7], the low-risk group represented 11.5% of the whole series of patients with nonrecurrent, nonmuscle-invasive bladder tumors. Although there are demographic differences between European countries and other worldwide countries, the low-risk group of nonmuscle-invasive bladder tumors is a very frequent entity in the uro-oncology field.

## DIAGNOSIS

Cystoscopy and cytology are the mainstream methods for the diagnosis of bladder tumors, but the question is if there are reliable cystoscopic findings to identify low-risk tumors. In two studies, Herr[11] correlated the cystoscopic appearance of bladder tumors and their pathological examination. Patients with tumors <0.5 cm, with discrete papillary fronds and a visible fibrovascular core were considered as TaG1 tumors. In a retrospective study, 78 (93%) of those considered cystoscopically TaG1 were histologically proven; when urinary cytology was negative, the correlation was 99%. In this series, only 7% of low-grade tumors diagnosed cystoscopically were high grade and 2% of invasive tumors in pathological examination. These figures were also corroborated in a prospective study with 94 and 99% correlation between cystoscopy and histology in patients with TaG1 appearance and with negative urinary cytology, respectively[12].

In a randomized trial defining low-risk, nonmuscle-invasive bladder cancer, when papillary (thin fronds), <3 cm, single, primary, or recurrent with disease-free for more than 1 year, it was found that during the first cystocopic evaluation, 3% were invasive bladder tumors, 1.5% were G3, and 0.7% were bladder Tis[13]. With similar criteria, Oosterlink et al. observed that 5.6% of patients had muscle-invasive tumors[14].

Cina et al.[15], in nonselected patients, did not find a good correlation between cystoscopic appearance and histology; 54% of papillary tumors of high-grade appearance at biopsy were of low-grade appearance on cystocopy, 19% of those considered to be low grade on cystoscopy were high grade at biopsy, and 33% of high-grade appearance on cystocopy were low grade by pathological examination.

The discrepancy between this and previous series was probably due to the strict selection criteria used in theformer series and the unselected character of patients included in the Cina et al. series[15]. According to these data, urinary cytology takes an important part in the bladder tumor assessment. A positive cytology excludes tumors at low risk and a negative cytology contributed to identifying tumors at low risk.

The need for intravenous urography at the initial diagnosis of bladder tumor is rather questionable, as the incidence of upper urinary tract tumors is extremely low, ranging from 0.3–2.3%[10]. A relation has been observed between grade and stage of bladder tumors and the incidence of upper urinary tumors: 0% for G1, 1.1% for G2, and 1.3% for G3; and 0% for Ta and 7% for T1[16]. In addition, the prognosis of patients with TaG1-2 does not seem to be threatened by the diagnosis delay of upper urinary tract tumors until they are symptomatic[17]. As a consequence, we can speculate that the incidence of upper urinary tract tumors in patients with low risk of nonmuscle-invasive bladder cancer is extremely low and a routine intravenous urography is not justified.

## TREATMENT

The standard treatment for patients with nonmuscle-invasive bladder cancer is the complete transurethral resection (TUR) of all bladder tumors. The TUR technique should be performed in fractionated fashion, removing the exophytic part first, the endophytic part (including muscularis propria) second, and the surrounding tissue of the tumor pedicle third. The inclusion of muscle in the second specimen is essential to carry out a correct staging. However, small tumors with clinical and cytological characteristics of low-risk tumor can be resected in only one chip, but the muscle inclusion in the chip is also important to establish the right stage.

Although residual tumors are related to the quality of the TUR[18], this is a very frequent event and also related to number, grade, and stage[19]. In two randomized trials with clinical criteria for low risk, a low recurrence rate (3–7%) was observed at the first cystoscopy in observation arms[13,14]. This suggests that the residual tumor in this group is not a relevant event and the indication of a second TUR 4–6 weeks after the initial TUR seems to be unrealistic.

Classically, the most common approaches for patients at low risk were observation alone or standard intravesical chemotherapy. With observation only, the recurrence rate is around 40%, meaning that a great number of TURs and endoscopic assessments should be performed in patients with no life-threatening disease according to the extremely low progression rate. Standard intravesical chemotherapy obviously reduces the recurrence rate in these patients, but it could be an overtreatment with a significant rate of side effects and high economic cost.

Between both approaches, an immediate single instillation of a chemotherapy agent was prompted according to the results of four phase II trials, which suggests that an immediate single instillation of intravesical chemotherapy after TUR reduced the recurrence rate[19,20,21,22,23]. These preliminary results encouraged an undertaking of randomized trials in order to demonstrate the effectiveness of this approach.

The Medical Research Council[24] carried out a phase III trial that compared a single instillation of thio-tepa, a 3-month instillation for 1 year, and TUR alone. No significant difference of recurrence rate was reported between the three arms. Afterwards, another large randomized trial from EORTC, with a mean follow-up of 2 years, a significant reduction of recurrence rate, and the interval to the first recurrence, was found in patients treated with a single immediate instillation of epirubicin compared to TUR plus water[14]. Five other randomized trials showed a significant reduction of recurrence rate in patients receiving a single immediate instillation of chemotherapy agents compared to those included in the controlled arm (Table 1). Evaluating the efficacy of this approach, a 12% recurrence rate reduction was observed in patients treated with active agents compared to the control group.

**TABLE 1**
**Randomized Trials Comparing Immediate Intravesical Chemotherapy and Control Arms**

| Author (Year) | Patients | Timing | Agent | Recurrence Rate | |
|---|---|---|---|---|---|
| | | | | RTU (Agent) | RTU (Control) |
| Oosterlink et al. (93) | 399 | 6 h | Epirubicin | 28.3% | 37.5% |
| MRC (95) | 379 | Unknown | Thiotepa | 37.3% | 41.9% |
| Tolley et al. (96) | 306 | 24 h | Mitpmycin C | 48.3% | 59.8% |
| Ali-El-Dein et al. (97) | 109 | Immediate | Epirubicin | 23.6% | 51.8% |
| Solsona et al. (99) | 131 | 6 h | Mitomycin C | 40.3% | 54.6% |
| Rajala et al.(02) | 134 | Immediate | Epirubicin | 45.5% | 72.7% |
| Okamura et al. (02) | 160 | 6 h | Pirarubicin | 25.9% | 43% |
| Total | 1618 | | | 36.7% | 48.4% |

In trials using a single immediate instillation, different chemotherapy agents were used with similar efficacy except for thio-tepa. Nevertheless, in other two trials[20,29] using higher doses of thio-tepa (60 and 90 mg.) than the MRC trial (30 mg), the recurrence rate was reduced in patients receiving thio-tepa. As a whole, 50.5% of recurrence was observed in the control arm and 35.6% in patients treated with a single instillation of a chemotherapy agent with epirubicin, mitomicin C (MMC), or pirarubicin.

In a recent meta-analysis, Sylvester et al.[30] analyzed seven randomized trials, which included at least one group with only one immediate instillation of a chemotherapic agent. Among 1476 patients included in this meta-analysis, with a median follow-up of 3.4 years, 362 of 748 (48.4%) of patients treated with TUR alone had a recurrence, whereas 267 of 728 (36.7%) of those receiving a single instillation of a chemotherapy agent had a recurrence. This difference means a significant decrease in the risk of recurrence of 39% (odds ratio of 0.61; $p < 0.0001$). This positive impact on recurrence is observed for patients with solitary tumors (odds ratio of 0.61) as well as for multiple tumors (odds ratio of 0.44); although the significant reduction of recurrence in patients with multiple tumors when receiving a single immediate instillation the recurrence rate was very high, 65.2%. This high recurrence means that a single immediate instillation, even though positive, is an insufficient approach for these patients needing more instillations. Moreover, in other randomized trials not included in this meta-analysis, by administrating more than one single instillation, it was demonstrated that an early instillation was also effective in patients with multiple tumors than those not receiving an immediate instillation[29,31].

One controversial issue is the timing of the instillation. As a whole, the instillation was generally administrated immediately or within 6 h after the TUR. This seems a suitable period to achieve the highest effectiveness. Whether or not the chemotherapy instillation is effective after this period remains unknown. However, Kaasinen et al.[32] analyzed a randomized series of patients receiving an intravesical instillation after 24 h because of protocol deviation and found that the recurrence rate was almost double that for those who received the instillation within 24 h after TUR. Although the suitable period seems to be within 6 h, this might be enlarged to 24 h, but no longer.

The role of bacillus Calmette-Guerin (BCG) in the low-risk group is poorly understood as, in general, BCG is not used for these patients. However, in two meta-analyses, BCG was not superior to MMC in low-intermediate risk groups[33,34]. Only when maintenance was applied was BCG superior to MMC[33], however, nonrandomized trials were included in this stratification of the meta-analyses and some methodological problems make this evidence very weak. In another meta-analysis, BCG was only superior to intravesical chemotherapy when this approach was applied after intravesical chemotherapy failed[35]. On the other hand, in all meta-analyses, BCG was significantly more toxic than intravesical chemotherapy regardless of the scheme used. In consequence, BCG seems not to have a role as an initial treatment in the low-risk group, but probably could be effective as a rescue therapy when intravesical therapy fails.

## MECHANISM OF ACTION

Most of the results of trials using a single immediate instillation were analyzed in short-term follow-up. The impact of this approach in long-term follow-up was analyzed by Solsona et al.[13] who compared a single instillation of MMC and TUR alone. With a median follow-up of 94 months, the recurrence rate was dramatically reduced during the first months, reaching the statistical significance at 24 months evaluation ($p = 0.013$), and this difference was maintained during the follow-up according to the Kaplan Meier method in which curves run parallel (Fig. 1). Rajala et al.[26], with a median follow-up of 72 months comparing a single instillation of epirubicin, also observed that curves run parallel during the follow-up after an initial separation. Both series confirm that the efficacy of an immediate instillation is concentrated during a short interval from the instillation and, after this period, the natural history was not modified by this approach. The critical period of this approach was also studied by Solsona et al. who reported that this period was concentrated during the first year (Fig. 2), with a recurrence rate of 3.5% in the immediate instillation arm and 20.3% in the observation arm ($p < 0.05$). However, after 12 months, the recurrence rate of both approaches was similar, 36.8 and 32.8%, respectively. According to these observations, one can speculate that the working mechanism of an immediate instillation is to eliminate floating tumor cells, preventing the cells' implantation at traumatized bladder mucosa after TUR. In animal models, a potential cell implantation as responsible for early recurrence in the bladder was demonstrated, as well as the inhibition of implantation cells by thio-tepa in cauterized bladder mucosa[36]. Also, a potential chemoresection of residual tumors has been suggested. Masters et al.[37] observed a 44% marker lesion disappearance after a single instillation of epirubicin. However, that a single dose is able to achieve the complete elimination of residual tumor needs more scientific support.

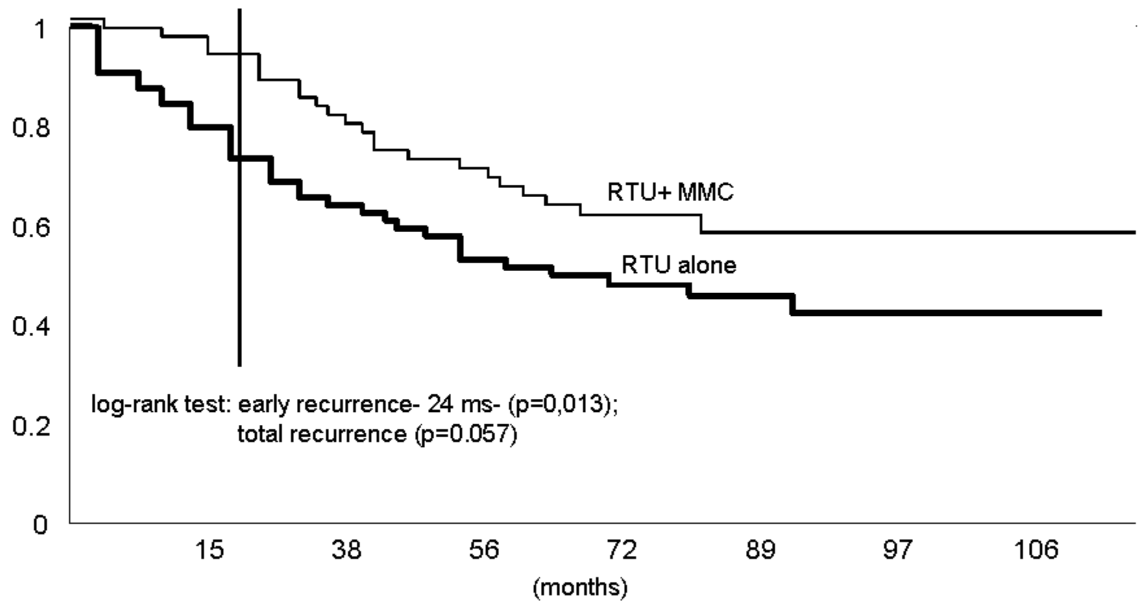

**FIGURE 1.** Recurrence-free survival comparing a single immediate chemotherapy instillation and observation. Short- and long-term follow-up.

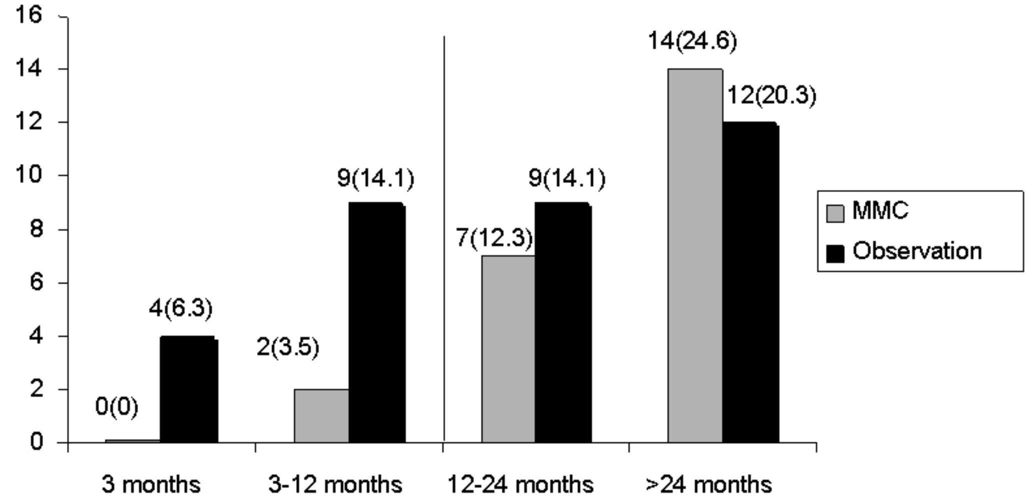

**FIGURE 2.** Recurrence percentage comparing a single immediate chemotherapy instillation and observation. Time of recurrence.

Oosterlink et al.[10] calculated that the NNT (number needed to treat) to prevent one recurrence was 8.5, meaning a cost reduction in terms of surgery and hospitalization.

## TOXICITY

The tolerance of an immediate instillation is usually excellent with minimal side effects. In the Solsona et al. series, a 3.5% slight chemical cystitis and skin reactions were observed in the MMC arm and only 1.5% in the observation arm[13]. This minimal side effect was also reported in the meta-analysis carried out by Sylvester et al.[30]. However, severe side effects have been published, particularly chemical pericystitis related to drug extravasation due to bladder perforation[38]. Although these severe complications are unusual, some precautions should be adopted when a bladder perforation is suspected during TUR. In these cases, an immediate instillation should not be administrated. In cases of hematuria, the instillation should be delayed until the cessation of the hematuria, due to potential urinary retention and urine extravasation.

## FOLLOW-UP

Regardless of the good prognosis for patients included in the low-risk group, they should be followed according to the high recurrence rate. However, early detection should not be as important as in intermediate- or high-risk groups since the recurrences usually are the same category than the initial pathology and the progression rate is extremely low[11]. During the follow-up, patients with TaG1 tumors who progressed to higher grade ranged from 13.4–16.3%[39,40], only 3.3% to T1, and from 1.1–3.3% to muscle-invasive bladder tumor. In a series of a clinical low-risk group with a median follow-up of 94 months, only 1.5% developed progression to invasive tumors[13]. Soloway et al.[41] observed a growth of 1.77 mm/month in 32 patients with low-grade tumors only. Among these patients, only three increased to a higher grade or stage, meaning that the delayed TUR does not threaten the prognosis of these patients. According to these data, the follow-up schedule should include the cystoscopy and urinary cytology at 3 months as the great prognostic value of this evaluation[10] and if this is negative, the cystoscopy can be spared for 9 months followiing Oge et al.'s proposal[42]. The duration of the follow-up should be indefinite as the risk of recurrence remains life-long. However, many cystosocpies should be replaced by ultrasounds and cytology according to expert opinion. Regardless, the high sensitivity of urinary markers, whether they can replace cystoscopy, remains to be defined as there is no scientific evidence proving their efficacy in this group within the balance of cost/efficacy[10].

In case of recurrence, the best therapeutic approach remains controversial. Herr[11], based on the accuracy of cystoscopy appearance in patients with recurrent TaG1 and negative cytology, proposed only fulguration in the office. Of 69 patients who underwent office fulguration, 22 required TUR and 3 had muscle-invasive tumors. In a prospective study from the same institution, of 267 consecutive patients with recurrent tumors, 103 underwent only fulguration because they had low-grade appearance, <5 mm, and negative urinary cytology. There was no greater risk of progression in these patients than in those treated with TUR because they did not fulfill the prior criteria[10]. In summary, office fulguration seems to be an alternative to TUR in selected cases with recurrent TaG1 tumors.

An intravenous urography is not necessary during the follow-up as the recurrence in the upper urinary tract is extremely low in patients at low risk[10].

## CONCLUSIONS

Patients with nonmuscle-invasive bladder tumors at low risk constitute a group with high recurrence and low progression rates. A single immediate instillation of whatever chemotherapy agent should be considered as the best therapy option within the balance of cost/efficacy. This approach is useful for single and multiple tumors, but tumors with worse prognostic factors should be treated with more intensive intravesical chemotherapy, as the efficacy of this approach is limited to the first year and has no influence on the posterior natural history. Office fulguration is a suitable therapy in selected patients with recurrent tumors. BCG does not seem an appropriated treatment for these patients, but it might be considered as a rescue therapy when intravesical chemotherapy fails. Follow-up is necessary, but the schedule is less rigorous than for intermediate- or high-risk groups.
